# Dietary Acid Load and Mental Health Outcomes in Children and Adolescents: Results from the GINIplus and LISA Birth Cohort Studies

**DOI:** 10.3390/nu10050582

**Published:** 2018-05-08

**Authors:** Judith Bühlmeier, Carla Harris, Sibylle Koletzko, Irina Lehmann, Carl-Peter Bauer, Tamara Schikowski, Andrea von Berg, Dietrich Berdel, Joachim Heinrich, Johannes Hebebrand, Manuel Föcker, Marie Standl, Lars Libuda

**Affiliations:** 1 Department of Child and Adolescent Psychiatry, University Hospital Essen, University of Duisburg-Essen, 45147 Essen, Germany; judith.buehlmeier@uni-due.de (J.B.); johannes.hebebrand@uni-due.de (Joh.H.); manuel.foecker@uni-due.de (M.F.); 2 Dr. von Hauner Children’s Hospital, University Hospital, LMU of Munich, 80337 Munich, Germany; carla.harris@helmholtz-muenchen.de (C.H.); sibylle.koletzko@med.uni-muenchen.de (S.K.); 3 Institute of Epidemiology, Helmholtz Zentrum München—German Research Centre for Environmental Health, 85764 Neuherberg, Germany; Joachim.heinrich@med.uni-muenchen.de (Joa.H.); marie.standl@helmholtz-muenchen.de (M.S.); 4 Department of Environmental Immunology/Core Facility Studies, Helmholtz Centre for Environmental Research—UFZ, 04318 Leipzig, Germany; irina.lehmann@ufz.de; 5 Charitè—Universitätsmedizin Berlin, 10117 Berlin, Germany; 6 Berlin Institute of Health, 10178 Berlin, Germany; 7 Department of Pediatrics, Technical University of Munich, 80804 Munich, Germany; carl-peter.bauer@drv-bayernsued.de; 8 IUF—Leibniz Research Institute for Environmental Medicine, 40225 Düsseldorf, Germany; tamara.schikowski@IUF-Duesseldorf.de; 9 Research Institute, Department of Pediatrics, Marien-Hospital Wesel, 46483 Wesel, Germany; avb.rodehorst@gmx.de (A.v.B.); berdel.vonberg@t-online.de (D.B.); 10 Institute and Outpatient Clinic for Occupational, Social and Environmental Medicine, Inner City Clinic, University Hospital of Munich (LMU), 80336 Munich, Germany; 11 Allergy and Lung Health Unit, Melbourne School of Population and Global Health, The University of Melbourne, Victoria 3010, Australia

**Keywords:** PRAL, acid base balance, low grade metabolic acidosis, SDQ, emotional problems, hyperactivity

## Abstract

High dietary acid load may have detrimental effects on mental health during childhood and adolescence. We examined cross-sectional and prospective associations of dietary acid load and mental health problems in a population-based sample, using data from the German birth cohort studies GINIplus (German Infant Nutritional Intervention plus environmental and genetic influences on allergy development) and LISA (Influences of lifestyle-related factors on the immune system and the development of allergies in childhood). These studies included detailed assessments of dietary intake through a food frequency questionnaire (FFQ), mental health outcomes measured through the Strengths and Difficulties Questionnaire (SDQ), and covariates. Using logistic regression, cross-sectional associations between dietary acid load measured as potential renal acid load (PRAL) and SDQ subscales were assessed at age 10 years (*N* = 2350) and 15 years (*N* = 2061). Prospective associations were assessed, considering PRAL at 10 years as exposure and SDQ subscales at 15 years as outcome (*N* = 1685). Results indicate that children with a diet higher in PRAL have more emotional problems (OR = 1.33 (95% CI = 1.15; 1.54); *p* < 0.001), and show hyperactivity more often (1.22 (1.04; 1.43); *p* = 0.014) at 10 years. No significant associations were present either cross-sectionally at age 15 years, nor prospectively. Results were confirmed in sensitivity analyses. These findings reveal first evidence for potential relationships between PRAL and mental health in childhood, although we cannot exclude reverse causality, i.e., that dietary behavior and PRAL are influenced by mental status. Future studies should address confirmation and identify biological mechanisms.

## 1. Introduction

According to the World Health Organization (WHO), mental disorders are a growing burden worldwide, and many of them begin in childhood or early adolescence [1]. In Germany, around 15% of children and adolescents are currently afflicted by at least one mental health problem that affects their health-related quality of life [2]; manifestations may have lifelong consequences. The identification of targets for prevention strategies is urgently needed to reduce incidences. During recent years, growing evidence has indicated that besides psychological, social, and environmental factors, diet composition also constitutes a modifiable factor [3,4].

For depressive symptoms there are several prospective studies showing inverse associations with high quality, health-associated dietary patterns, such as Mediterranean or vegetarian-based diets [5]. Although biological pathways involving changes in inflammation status—i.e., derived by changes in n-3/n-6 polyunsaturated fatty acids (PUFA) intake [6]—are under discussion, up to now, these have been poorly understood [7]. In addition, observational studies [8,9,10] in habitual vegetarian eaters indicate that this pattern typically contains more alkali-rich food groups such as fruit and vegetables, and less high-phosphorus, high-protein foods, because the intake of animal products is limited [11]. The amounts of consumed phosphorus-rich and sulfur-rich food items on one hand (potential anorganic acid precursors) and of alkali-rich food items on the other (potential bicarbonate precursors) majorly determine endogenous acid and base production, respectively [12]. Thus, Deriemaeker et al. [8] found significantly lower dietary acid loads in vegetarian compared to omnivore diets (potential renal acid load(PRAL): −11 ± 20 mEq/d vs. 14 ± 17 mEq/d; *p* < 0.001). Consistently, the acid load of a Mediterranean diet was revealed to lie between those of vegetarian and omnivore diets (mean PRAL: −2 mEq/d (95%CI −5; 2) [13]), as it is characterized by mainly, but not exclusively, plant-based meals. In contrast to those net-base producing diets, net-acid producing diets constitute a moderate, but continuous demand of the body’s buffer systems, and are thus discussed as one cause of chronic diseases such as osteoporosis and cardiovascular disease [14,15].

For the etiology of mental diseases, the net base-forming characteristic of the described high-quality dietary patterns may constitute another protective factor besides known anti-inflammatory substances. Recent findings suggest that even moderate increases in dietary acid load relevantly stimulate the secretion and activity of glucocorticoids (GC) [16,17], which play a central role in body–brain interaction. In a sample of 200 healthy children, those with higher dietary acid loads (PRAL: −8 ± 11 vs. 17 ± 10 mEq/d; *p* < 0.0001) secreted more GCs (*p* = 0.04) and had more potentially bioactive GCs available (*p* = 0.009) [16]. This is to be expected, since GCs are known to increase the organism’s capacity to excrete acid [18,19]. However, with long-term exposure to high dietary acid loads, an overall higher GC activity seems to cause adverse somatic effects [20,21,22]. For example, in a cross-sectional study in >400 overweight children and adolescents, those in the highest cortisol tertile (>384 nmol/L) had significantly increased odds of hypertension (>95th percentile; odds ratios (OR) 1.593; 95% CI 1.002–3.133) in univariate and multivariate models [22]. Effects on mental health are also plausible, since GCs cause changes in limbic areas of the brain (prefrontal cortex, amygdala, and hippocampus), and thereby modulate emotional evaluation and behavior [23,24]. Hypercortisolism in Cushing’s disease, for instance, induces hippocampus volume reductions and neuropsychological impairment [25].

Detrimental effects of a high dietary acid load on mental health because of increasing GC activity might become apparent especially during childhood and adolescence, as the brain is particularly vulnerable during this developmental stage. This may be true for short-term effects as well as in the long run. In an explorative approach, we therefore aimed at examining cross-sectional and prospective associations of dietary acid load and the risk for mental health problems in a population-based sample of children and adolescents.

## 2. Materials and Methods

### 2.1. Study Population

For the present analyses, data from the ongoing German birth cohort studies GINIplus and LISA were combined. The GINIplus study (German Infant Nutritional Intervention plus environmental and genetic influences on allergy development) included 5991 healthy newborns from urban Munich and rural Wesel; families were recruited between September 1995 and June 1998. The LISA cohort (Influences of lifestyle-related factors on the immune system and the development of allergies in childhood) included 3094 healthy newborns; families were recruited between November 1997 and January 1999 from Munich, Wesel, Leipzig, and Bad Honnef. Details on the study designs and subject recruitment have been described previously [26,27]. Both studies were conducted in accordance with the ethical standards laid down in the Declaration of Helsinki and approved by the local ethics committees (Bavarian General Medical Council, University of Leipzig, Medical Council of North Rhine-Westphalia). All of the participants’ families gave written informed consent before study onset. For the present examination, we focused on the assessments at age 10 and 15 years, since the study schedule at this age included the assessment of detailed information on dietary intake, mental health outcomes, and covariates (by questionnaires or during medical examinations).

### 2.2. Dietary Assessment and Estimation of Diet-Induced Acid Load

A self-administered food frequency questionnaire (FFQ), which was developed to assess habitual dietary intake during the past 12 months prior to its completion, was administered once at the 10-year follow-up and once at the 15-year follow-up. The questionnaire was specifically designed and validated to estimate intakes of energy, fatty acids, and antioxidants in 10-year-old children [28]. Details on the FFQ validation, implementation, and data extraction have been described previously [28,29,30]. Briefly, the FFQ contained questions on the intake of 80 food items. The participants were asked to choose one of nine frequency categories (“never” to “more than four times a day”), and for estimation of quantities, common portion sizes for each food item were assigned. For food items that are difficult to describe in common household measures, coloured photographs from the EPIC (European Prospective Investigation into Cancer and Nutrition) study showing three different portion sizes were provided [31]. Specific questions concerning preferred energy and fat content, preparation methods, and supplement use were also included. At the 10-year follow-up, parents completed the FFQ alongside their children, whereas at 15 years, the FFQ addressed participants directly. A standardized quality control was applied based on recommendations for data cleaning in nutritional epidemiology [32], which has been described in detail elsewhere [30]. Consumption frequency and estimated portion sizes were converted into average daily intakes (g/d), and the corresponding energy and nutrient contents were calculated based on the German Food Code and Nutrient Database (BLS), version II.3.1, 2005 (Federal Research Center for Nutrition and Food (BfEL), Karlsruhe, Germany).

The dietary component of endogenous acid production was estimated according to the PRAL (potential renal acid load) model by Remer and Manz [11,33]. The model is based on acid base physiology and the contribution of anorganic acids and bicarbonate to acid base balance [12]. It takes the nutritional intake of mineral cations (potential bicarbonate precursors), anorganic anions, and protein (potential acid precursors), as well as their average absorption into account (PRAL < 0: dietary excess of base precursors; PRAL > 0: dietary excess of acid precursors). The model has been validated in adults [11] and children [33] by estimating renal net acid excretion. Individual PRAL values were calculated using the following formula:

PRAL [mEq/d] = 0.49 × protein intake [g/d] + 0.037 × phosphorus intake − 0.021 × potassium [mg/d] − 0.026 × magnesium [mg/d] − 0.013 calcium [mg/d](1)

The distribution of PRAL in females and males at the 10-year and 15-year follow-ups is presented by boxplots in the Supplementary Figure S1. Participants presenting extreme PRAL values were visually identified and distinguished in the boxplots by dotted lines. In order to avoid effects being driven by a few extreme cases rather than by the overall data, these were considered as outliers, and excluded from the analyses (Figure S1). At age 10 years, this comprised females with PRAL values outside the range −28 to 46 and males outside −34 to 53; and at age 15 years, females with values outside the range −38 to 73, and males outside −40 and 106. The coefficient of the correlation between PRAL reported at the 10-year follow-up and PRAL at the 15-year follow-up was 0.3, as assessed by Pearson’s product-moment correlation in participants with PRAL data available at both time points.

### 2.3. Measurement of Mental Health Outcomes

Mental health problems were assessed by the Strengths and Difficulties Questionnaire (SDQ) [34]. The SDQ is a brief screening instrument containing 25 items divided into five subscales (5 × 5, emotional problems, conduct problems, hyperactivity–inattention, peer problems, and prosocial behavior). Each item can be marked on a three-point Likert scale (0 = not true, 1 = somewhat true, 2 = certainly true). The scores of each item are summed up to a total subscale score (0–10). The scores of the four subscales that would generally be thought of as difficulties (emotional problems, conduct problems, hyperactivity–inattention, peer problems) generate a total difficulties score ranging from 0–40. Information on mental health obtained at age 10 years was based on a parent-completed SDQ, whereas at age 15 years, participants self-rated their problems. Given the highly skewed distribution of the SDQ scores, these were categorized into three levels indicating a “normal”, “borderline”, or “abnormal” degree of difficulties. Scores at age 10 years were categorized based on existing German cut-off recommendations for the parent-reported SDQ [35,36]. Since no official German cut-off recommendations exist for the self-rated version of the SDQ, scores obtained at the 15-year follow-up were categorized based on the widely-used British cut-offs [34]. For statistical analyses, a dichotomous variable was created for each SDQ subscale, in which the “normal” respondents were compared to “borderline/abnormal” respondents. This approach was chosen in order to facilitate interpretation, and to avoid reporting heterogeneity bias [37]. In sensitivity analyses, we examined the impact of grouping the “borderline” respondents together with the “normal” respondents (i.e., modeling “normal/borderline” versus “abnormal”).

### 2.4. Covariates

The present analyses were adjusted for a number of covariates and potential confounders. Firstly, variables that are specific to the study design of the GINIplus and LISA cohorts were included in the model as covariates. These were namely the study (GINIplus observation arm; GINIplus intervention arm; LISA) and recruitment region (Munich; Leipzig; Bad Honnef; Wesel). Both variables determine the general characteristics of the study population, which could be relevant in the association between PRAL and mental health outcomes. Further covariates were selected a priori on the basis of known associations with SDQ outcomes and PRAL. Information on the selected covariates was obtained through questionnaires or during medical examinations, and included the following: sex (male; female); total energy intake, estimated from the FFQ (kcal/day); sedentary behavior, as defined by reported daily hours spent in front of a screen (low: ≤2 h/day; high: >2 h/day); moderate-to-vigorous physical activity (MVPA), defined as weekly hours of exercise performed outside school, with exercise constituting any activity causing breathlessness or sweating (grouped based on distribution within the study population as low: 25th percentile; medium: 25th–75th percentile; high: >75th percentile); body mass index (BMI), calculated from measured or reported weight and height (kg/m^2^); pubertal status, defined at age 10 years as pubertal onset (yes: parents stated the presence of acne or spots, pubic or axillary hair, breast development, menstruation, penis or testicle enlargement), and at age 15 years as pubertal stage based on a self-rating pubertal development scale (pre, early, mid, late, or post-pubertal); parental education defined by the highest grade completed by either the mother or the father on the basis of the German educational system (low/medium: ≤10th grade; high: >10th grade); parental psychopathology, defined as cases with a Global Severity Index score (from the Brief Symptom Inventory 18 [38]) greater than the 90th percentile in order to ensure a sufficient number of cases, as there are no published reference values for the German population (yes; no). Information on most of the covariates was obtained at the respective 10 and/or 15-year follow-ups, with the exception of study, region, and sex (recorded upon recruitment at birth), parental education (reported in earlier follow-up questionnaires nearer to the studies’ commencement), and parental psychopathology (reported at the 15-year follow-up). Most of the covariates that were selected a priori qualified as confounders by definition, following bivariate analyses of associations with both the exposure and outcomes of interest. Exceptions were MVPA and parental psychopathology, which were however strongly associated with SDQ outcomes. In order to ensure an accurate estimate, we included MVPA in the model along with all other confounders. Adjustment for parental psychopathology, along with the above-mentioned covariates, was performed in sensitivity analyses in an attempt to maintain the largest-possible sample size and power in the main analyses. Further sensitivity analyses excluded children who presented any form of chronic disease that was likely to influence mental health and/or diet (celiac disease, diabetes, or cancer). Finally, to assess the robustness of our findings in the context of other relevant dietary aspects, we considered the possibility of confounding by long chain n-3 PUFA, total n-3 PUFA, and total n-6 PUFA, estimated from the FFQ (% of total energy intake). Total n-3 PUFA and n-6 PUFA fulfilled the criteria as potential confounders. Given the abundance of literature associating n-3 PUFA with mental health outcomes [3,39], we opted for a sensitivity analysis adjusting for n-3 PUFA (not n-6 PUFA, as these variables are highly correlated).

### 2.5. Statistical Analysis

Subject characteristics in the total population and stratified by sex were described at ages 10 and 15 years by medians (25th percentile; 75th percentile) for continuous variables, or counts (%) for categorical variables. Differences between females and males were tested by Wilcoxon’s rank sum test for continuous variables, and by Pearson’s χ^2^ test for categorical variables. Using logistic regression, cross-sectional associations between diet-induced acid load and total difficulties, as well as each mental health subscale (emotional problems, conduct problems, hyperactivity–inattention, peer problems, and prosocial behavior) were assessed at age 10 years (*N* = 2350) and at age 15 years (*N* = 2061). Each cross-sectional model was adjusted for sex, total energy intake, sedentary behavior, moderate-to-vigorous physical activity, body mass index, pubertal status, parental education, study, and recruitment region. Prospective associations were then assessed, considering PRAL at age 10 years in relation to each mental health subscale at age 15 years (*N* = 1685). Regression models assessing prospective associations were further adjusted for the respective mental health outcome reported at age 10 years. For example, if testing the effect of PRAL intake at age 10 years on total difficulties categorized as “normal” versus “borderline/abnormal” at age 15 years, the model was further adjusted for total difficulties at age 10-years, categorized in the same way.

Results of the described cross-sectional and prospective logistic regression analyses are presented as odds ratios (OR) and 95% confidence intervals (95% CI), and modeled per interquartile range (IQR) increase in PRAL (IQR in 10-year cross-sectional analyses sample = 14.9 mEq/d; IQR in 15-year cross-sectional analyses sample = 17.8 mEq/d; IQR in prospective analyses sample = 14.7 mEq/d). Given the differences in PRAL values observed between females and males, we tested for possible interactions with sex by including an interaction term between PRAL and sex. No significant interaction was observed, and so no further sex-stratified analyses were performed. The same analyses as described above were then carried out using the more stringent binary definitions for SDQ subscales: “normal/borderline” versus “abnormal”.

A number of further sensitivity analyses were performed to confirm the robustness of our findings. First, the above analyses were re-run with the models additionally adjusted for parental psychopathology. Further sensitivity analyses excluded participants who reported suffering from a chronic disease (18, 19, and 16 subjects excluded from the 10-year cross-sectional, 15-year cross-sectional, and prospective analyses, respectively). Finally, analyses were run with the models additionally adjusted for n-3 PUFA. In further sensitivity analyses, to allow for a sound comparison of results from cross-sectional and prospective analyses, the sample was limited to participants with complete dietary and SDQ data at both 10 and 15 years (*N* = 1146; PRAL IQR at age 10 years = 14.2 mEq/d; PRAL IQR at age 15 years = 16.8 mEq/d). Suggested cut-offs for the categorization of the German self-report version of the SDQ have been published by Lohbeck et al. [40]. In order to check the consistency of our findings when applying different choices of cut-offs, additional sensitivity analyses were carried out using the alternative German cut-off values (only 15-year cross-sectional and prospective analyses, first including maximal available data, and then limited to participants with complete data at both time points). All of the analyses were conducted using R, version 3.3.2 [41]. Logistic regression was calculated using the “glm” function.

## 3. Results

### 3.1. Study Population

**Figure 1:**
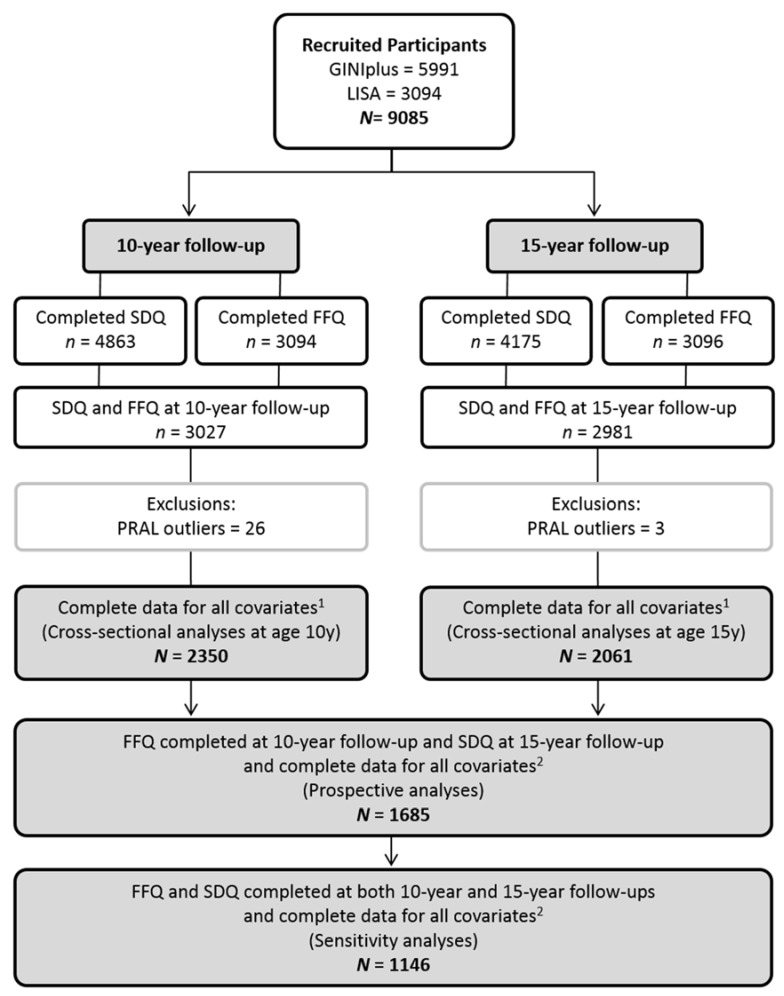
Flowchart study population. Potential renal acid load (PRAL) outliers, at age 10 years: females with values outside the range −28 to 46, and males outside −34 to 53; at age 15 years: females with values outside the range −38 to 73, and males outside −40 and 106; ^1^ Covariates: total energy intake, body mass index, moderate–vigorous activity, screen time, pubertal status, parenteral education, study (arm), region, sex; ^2^ Additional covariate: Strengths and Difficulties Questionnaire (SDQ) at age 10 years.

An overview of the derived study population for cross-sectional and prospective analyses is provided in Figure 1. Following the exclusion of outliers, complete data for cross-sectional analyses was available for 2350 participants at age 10 years, and 2061 participants at age 15 years. Prospective analyses included 1685 participants. Sensitivity analyses, which were limited to complete data for SDQ and FFQ at age 10 and 15 years, included 1146 participants.

Descriptive characteristics of the study population at the 10-year and 15-year follow-ups are displayed in Table 1. At age 10 years, males presented a higher prevalence of total difficulties, and specifically, of conduct problems, hyperactivity, and peer problems than females. Prosocial behavior was also more prevalent in males. At age 15 years, similar differences were observed, except females presented a higher prevalence of emotional problems, and total difficulties were equally prevalent in both sexes. Average PRAL levels were greater at age 15 years, with males presenting higher levels than females at both ages. Descriptive characteristics of the study population with complete data for SDQ and FFQ at age 10 and 15 years, included in sensitivity analyses, are provided in Supplementary Table S6 (*N* = 1146).

### 3.2. Association between PRAL and SDQ—Main Analyses

Results from cross-sectional analyses, where cases were defined as “borderline/abnormal”, indicated significant positive associations of PRAL with emotional problems (OR = 1.33 (95% CI = 1.15; 1.54)) and with hyperactivity (1.22 (1.04; 1.43)) at age 10 years (Table 2: A). With more stringent criteria for cases defined as “abnormal”, a significant association was further observed with total difficulties (1.25 (1.02; 1.53)) (Table 2: B). No significant associations were present either cross-sectionally at age 15 years, nor prospectively, in analysis including maximal available data at each follow-up (Table 2).

### 3.3. Sensitivity Analyses

Additional adjustment of the analyses for parental psychopathology did not drastically alter the main findings, although stronger associations were generally present. Furthermore, a significant cross-sectional association with total difficulties was observed at age 10 years (“borderline/abnormal” versus “normal”), which was not significant in the main analyses (Table S1).

In further sensitivity analyses, excluding participants with chronic diseases did not significantly alter the above-described associations (Table S2). Additionally, adjusting the analyses for n-3 PUFA did not substantially affect the main findings (Table S3), although the association with total difficulties at age 10 years (“abnormal” versus “borderline/normal”) was no longer significant (*p* = 0.051).

In sensitivity analyses including only participants with complete dietary and mental health data at both 10 years and 15 years (*N* = 1146), only the cross-sectional association between PRAL and emotional problems defined as “borderline/abnormal” remained statistically significant at age 10 years (1.39 (1.12;1.71)) (Table S4: (A)). When considering the more stringent definition of cases, a significant cross-sectional association was observed with total difficulties at age 15 years (2.35 (1.03; 5.37)), and a direct association with hyperactivity was observed in the prospective analyses (1.64 (1.12; 2.39)) (Table S4: (B)).

In additional analyses using the alternative German cut-off values for the self-rated version of the SDQ suggested by Lohbeck et al. [40], results were similar to those obtained with the British cut-offs [34], with no significant associations observed either cross-sectionally at age 15 years, nor prospectively (Table S5: 1). For analyses limited to participants with complete data at both 10 and 15 years, the association between PRAL and total difficulties at age 15 years was no longer significant, but a significant prospective association was still observed with hyperactivity (1.64 (1.12; 2.39)) (Table S5: 2).

## 4. Discussion

To the best of our knowledge, the present study is the first to examine the relationship between the acid load of the habitual diet of children and adolescents and their mental health status, using data from large-scale population-based samples. The main analyses show that higher dietary acid loads are associated with greater odds of emotional problems and hyperactivity in children at the age of 10 years, while no cross-sectional or prospective associations were observed in adolescents. In 10-year-olds, an increase in dietary acid load of 15 mEq/d was associated with 33% higher odds of emotional problems such as having fears and worries, a downhearted mood, and weak self-confidence, and 22% higher odds of hyperactivity referring to activity levels, attention, and impulse control. These results were in general confirmed in several sensitivity analyses. A more stringent binary definition of SDQ subscales, i.e., a comparison between “normal/borderline” versus “abnormal” even revealed positive associations with the total difficulty score. Interestingly, the odds for emotional problems and hyperactivity did not change substantially when cases were defined more strictly at abnormal values.

Due to the observational character of the study, we cannot conclude a causal relationship between dietary acid load and mental problems, especially since there are no previous studies that have examined this relationship. From very early studies, we know that systemic acid base balance modifies blood–brain transport [42] and glutamate turnover in the brain [43], thereby challenging homeostatic processes. In fact, in patients with chronical renal failure, the risk for depression increases proportionally to the degree of functional decline (and acid base disturbances) [44], but a first study did not relate laboratory markers of acid base balance to depression [45]. Accordingly, little insights on the relevance of acid base balance to mental health are deducible from states of severe metabolic acidosis.

One possible biologic mechanism for the potential effects of dietary acid load on mental health is given by earlier studies in healthy adults [17,46] and children from the DONALD study (DOrtmund Nutritional and Anthropometric Longitudinally Designed) [16], which have shown that already, variations in dietary acid load of 30 mEq/d lead to changes in GC activity. A recent study indicates that increased GC secretion already within the normal hormonal range has a somatic impact in healthy children [21]. Therefore, these magnitudes in GC activity might also be sufficient to affect brain structures, and thus emotions and behavior in childhood. The dietary acid load observed in our study was slightly to moderately acidifying. Although it was calculated based on nutritional assessment by the FFQ, which is an instrument that is not specially validated for intake of anions, cations, and protein, PRAL values were in a similar range (slightly/modestly acidifying) as earlier examinations from the DONALD study [47]. Thus, it is reasonable to consider the dietary acid load in the present study sufficient to stimulate GC activity. However, this hypothesis needs to be examined in future studies, as the present work did not assess GC data. In fact, due to the complexity of the calculation model, dietary PRAL might also have only indirect effects and act as a proxy for several varying food choices, which could also influence mental well-being. In German children, fruits, vegetables, and potatoes contribute significantly to dietary alkalinity, whereas animal and grain products add dietary acidity [47]. Thus, diets low in PRAL represent also a dietary pattern that is comparatively rich in fruits and (starchy) vegetables, which in general have been positively associated with mental health in children and adolescents [48,49,50]. Besides dietary alkalinity, fruit and vegetables contribute e.g., major amounts of flavonoids and (further) antioxidants to dietary intake, which could also be of relevance in the context of mental health processes. The positive influence of flavonoids on vascular [51,52] and cognitive function [53] is well documented; suggested pathways include the stimulation of (cerebral) blood flow. Impairments in cognitive function are also a common symptom in depression that majorly contributes to the persistence of the disease. However, since several sensitivity analyses (e.g., adjusting for n-3 PUFA intake) confirmed the results from the main analysis, this could indicate that PRAL might be directly related to mental health.

In a more strictly defined sample with complete data at 10 and 15 years (Table S4), we see a significant cross-sectional association between PRAL and total difficulties at age 15 years (abnormal versus borderline/normal). Given that very few adolescents were categorized as “abnormal” at the 15 year follow-up, we consider that this might represent a chance finding. In line with this, the main analysis shows no association between PRAL and any mental outcome in 15-year-olds, neither cross-sectionally nor prospectively. On the one hand, one could expect potential mental problems at this age to be strongly influenced by identity development and related conflicts so that dietary acid loading might play less of a role in neuropsychological modulation. On the other hand, we cannot exclude that these discrepancies underlie variation brought about by different informants reporting on mental health outcomes at the 10-year and 15-year follow-ups. At both ages, mental outcomes were assessed by the SDQ, which is a screening instrument that investigates whether a child/adolescent has significant emotional or behavioral problems, and the extent to which these impair social behavior, i.e., peer problems and prosocial behavior. At age 15 years, the participants themselves rated the SDQ, whereas at age 10, the parents were asked to rate their child’s mental health. The correlations between self-rated and parental rated mental health is generally only moderate; increasing age entails a reduced correlation particularly for internalizing problems such as emotional problems [54]. Consistently, prevalence rates for mental difficulties are lower compared with the German KIGGS cohort (German Health Interview and Examination Survey for Children and Adolescents) that assessed strengths and difficulties by parental rating (5.8% versus 17% (baseline survey) and 17.8% (wave 1) in total difficulties; borderline and abnormal values) [55].

Since this study is the first to analyze the association between PRAL and SDQ (sub)scores, an a priori definition of a primary outcome variable was not possible. This study must be understood as a first exploratory approach to give an impression of the relationship between PRAL and mental health outcomes. Our findings seem to indicate that future studies might primarily focus on associations between PRAL and emotional problems and/or hyperactivity in childhood, but it must be kept in mind that some of the few associations that were observed would no longer be significant if Bonferroni correction for multiple testing were to be conducted. More research is needed to confirm or refute a role of PRAL in regard to mental health.

The presented analyses cannot eliminate the possibility of associations arising due to reverse causation, especially since the prospective data in the main analysis do not show a relation between dietary acid load and mental problems. We know that mood states are a strong stimulus for food choices and that those who are more depressed make less health-related decisions [56], e.g., concerning fruit and vegetable intake. Net acid-forming patterns again may support—in a bidirectional manner—mental difficulties. Nonetheless, we do see a significant prospective association between PRAL and hyperactivity (abnormal versus borderline/normal) in a more strictly defined sample with complete data at 10 and 15 years (Table S4). Although one must be cautious with this outcome given the possible selection bias and the reduced sample, it might also be that this reflects particular characteristics of this subpopulation.

Besides the previously mentioned limitations, the FFQ as a dietary assessment tool is prone to measurement error. Although the FFQ was specifically designed and validated to assess habitual dietary intake in school-aged children (by parental report at the 10-year follow-up), and an extensive quality control was applied, the potential of measurement errors and a reduced ability to detect associations remains. Furthermore, the presence of residual confounding cannot be excluded due to the observational nature of the study. Overall, the underrepresentation of children from lower social classes in both study populations constitutes a non-random loss-to-follow-up, which may bias findings towards null, and thus to a certain extent limit the representation of the study area by the presented findings. However, despite the typically lower risk of mental health problems in populations of higher socio-economic status, significant associations were nevertheless detected, emphasizing the relevance of the studied topic and the need for further investigation. On the other hand, both studies offered the possibility of repeated measurement of diet and mental health in a large population sample of children/adolescents, allowing for prospective analyses. Also, the focus on healthy adolescents at a low risk of mental health problems provides a good setting for the detection of relevant risk factors for early preventive interventions.

## 5. Conclusions

This study revealed first indications for potential relationships between dietary acid load and mental problems. Due to the explorative character of the study, these findings need to be confirmed in future studies (observational and interventional studies). These should further examine the effects of extreme stages (i.e., acidosis) on mental health and the involved biological pathways. An increased intake of base-forming foods such as fruits and (starchy) vegetables and decreased intakes of acid-forming foods such as meat might represent one promising component of a global intervention strategy for the prevention of mental problems, especially in childhood.

## Figures and Tables

**Table 1:** Descriptive characteristics of study population at the 10-year and 15-year follow-up.

	10-Year Follow-Up				15-Year Follow-Up			
	Total (*N* = 2350)	Females (*N* = 1137)	Males (*N* = 1213)	*p*-Value ^a^	Total (*N* = 2061)	Females (*N* = 1101)	Males (*N* = 960)	*p*-Value ^a^
**Total difficulties** (borderline)	149 (6.34)	54 (4.75)	95 (7.83)	**<0.001**	99 (4.8)	60 (5.45)	39 (4.06)	0.110
(abnormal)	174 (7.4)	63 (5.54)	111 (9.15)		20 (0.97)	14 (1.27)	6 (0.62)	
**Emotional problems** (borderline)	174 (7.4)	86 (7.56)	88 (7.25)	0.910	63 (3.06)	49 (4.45)	14 (1.46)	**<0.001**
(abnormal)	223 (9.49)	110 (9.67)	113 (9.32)		71 (3.44)	64 (5.81)	7 (0.73)	
**Conduct problems** (borderline)	164 (6.98)	56 (4.93)	108 (8.9)	**<0.001**	78 (3.78)	37 (3.36)	41 (4.27)	**0.006**
(abnormal)	99 (4.21)	36 (3.17)	63 (5.19)		56 (2.72)	19 (1.73)	37 (3.85)	
**Hyperactivity** (borderline)	114 (4.85)	44 (3.87)	70 (5.77)	**<0.001**	134 (6.5)	68 (6.18)	66 (6.88)	**0.024**
(abnormal)	185 (7.87)	45 (3.96)	140 (11.54)		109 (5.29)	45 (4.09)	64 (6.67)	
**Peer problems** (borderline)	94 (4)	39 (3.43)	55 (4.53)	**0.045**	177 (8.59)	89 (8.08)	88 (9.17)	0.650
(abnormal)	108 (4.6)	42 (3.69)	66 (5.44)		37 (1.8)	19 (1.73)	18 (1.88)	
**Prosocial** (borderline)	109 (4.64)	32 (2.81)	77 (6.35)	**<0.001**	120 (5.82)	36 (3.27)	84 (8.75)	**<0.001**
(abnormal)	68 (2.89)	18 (1.58)	50 (4.12)		43 (2.09)	18 (1.63)	25 (2.6)	
**PRAL** (mEg/d) ^b^	6.63 (−0.36; 14.56)	4.96 (−1.47; 12.33)	8.43 (0.88; 16.12)	**<0.001**	9.39 (0.95; 18.72)	6.39 (−1.35; 13.88)	13.6 (5; 23.9)	**<0.001**
**BMI** (kg/m^2^) ^c^	16.6 (15.5; 18.4)	16.6 (15.5; 18.3)	16.6 (15.6; 18.4)	0.406	20 (18.6; 21.9)	20.1 (18.6; 21.8)	19.9 (18.4; 21.9)	0.114
**Total energy intake** (kcal/day) ^d^	1909 (1578; 2283)	1768 (1467; 2107)	2067 (1692; 2447)	**<0.001**	1979 (1538; 2511)	1712 (1362.; 2148)	2324 (1870; 2791)	**<0.001**
**Moderate–vigorous PA** (low)	544 (23.15)	322 (28.32)	222 (18.3)	**<0.001**	473 (22.95)	308 (27.97)	165 (17.19)	**<0.001**
(medium)	1262 (53.7)	607 (53.39)	655 (54.0)		1120 (54.34)	604 (54.86)	516 (53.75)	
(high)	544 (23.15)	208 (18.29)	336 (27.7)		468 (22.71)	189 (17.17)	279 (29.06)	
**Screen time** (high)	258 (10.98)	101 (8.88)	157 (12.94)	**0.002**	1099 (53.32)	509 (46.23)	590 (61.46)	**<0.001**
**Puberty signs** (yes)	674 (28.68)	536 (47.14)	138 (11.38)	**<0.001**	-	-	-	
**Pubertal stage** (pre–mid)	-	-	-		431 (20.91)	46 (4.18)	385 (40.1)	**<0.001**
(late)	-	-	-		1440 (69.87)	873 (79.29)	567 (59.06)	
(post)	-	-	-		190 (9.22)	182 (16.53)	8 (0.83)	
**Parental education** (high)	1628 (69.28)	816 (71.77)	812 (66.94)	**0.013**	1477 (71.66)	798 (72.48)	679 (70.73)	0.410
**Study (arm)** (GINI (observation))	853 (36.3)	434 (38.17)	419 (34.54)	0.084	756 (36.68)	419 (38.06)	337 (35.1)	**0.017**
(GINI (intervention))	628 (26.72)	307 (27)	321 (26.46)		528 (25.62)	298 (27.07)	230 (23.96)	
(LISA)	869 (36.98)	396 (34.83)	473 (38.99)		777 (37.7)	384 (34.88)	393 (40.94)	
**Region** (Munich)	1211 (51.53)	586 (51.54)	625 (51.53)	0.870	1129 (54.78)	583 (52.95)	546 (56.88)	0.061
(Leipzig)	185 (7.87)	86 (7.56)	99 (8.16)		169 (8.2)	82 (7.45)	87 (9.06)	
(Bad Honnef)	111 (4.72)	51 (4.49)	60 (4.95)		93 (4.51)	52 (4.72)	41 (4.27)	
(Wesel)	843 (35.87)	414 (36.41)	429 (35.37)		670 (32.51)	384 (34.88)	286 (29.79)	

Values are presented as counts (%) for categorical variables, and medians (25th; 75th percentile) for continuous variables. ^a^ Comparison between males and females: tested by Wilcoxon’s rank sum test for continuous variables, and by Pearson’s χ^2^ test for categorical variables (significant differences marked in bold: *p* < 0.05). ^b^ median (25th; 75th percentile) in subpopulation with no mental health problems: females age 10 years = 4.8 (−1.6; 12.3), males age 10 years = 8.4 (1.0; 15.8), females age 15 years = 6.4 (−1.3; 13.7), males age 15 years = 13.6 (5.1; 23.8). ^c^ median (25th; 75th percentile) in subpopulation with no mental health problems: females age 10 years = 16.6 (15.4; 18.2), males age 10 years = 16.6 (15.5; 18.3), females age 15 years = 20.1 (18.6; 21.8), males age 15 years = 19.9 (18.5; 21.9). ^d^ median (25th; 75th percentile) in subpopulation with no mental health problems: females age 10 years = 1774 (1477; 2115), males age 10 years = 2056 (1692; 2450), females age 15 years = 1712 (1352; 2142), males age 15 years = 2316 (1860; 2789). Moderate–vigorous PA = Moderate-to-vigorous physical activity (low: 25th percentile; medium: 25th–75th percentile; high: >75th percentile). GINI: German Infant Nutritional Intervention plus environmental and genetic influences on allergy development study; LISA: Influences of lifestyle-related factors on the immune system and the development of allergies in childhood.

**Table 2:** Odds ratio (OR) and 95% CI assessing the cross-sectional and prospective associations of diet-induced acid load (PRAL) with mental health outcomes ^a^.

	Cross-Sectional ^b^ (10-Year Follow-Up; *N* = 2350)		Cross-Sectional ^b^ (15-Year Follow-Up; *N* = 2061)		Prospective ^c^ (10- to 15-Year Follow-Up; *N* = 1685)	
	OR (95% CI)	*p*-Value	OR (95% CI)	*p*-Value	OR (95% CI)	*p*-Value
**(A) Borderline/abnormal vs. normal**						
Total difficulties	1.12 (0.96; 1.31)	0.139	1.02 (0.81; 1.28)	0.880	0.93 (0.72; 1.20)	0.566
Emotional problems	1.33 (1.15; 1.54)	**<0.001**	1.03 (0.81; 1.32)	0.805	1.02 (0.77; 1.34)	0.900
Conduct problems	0.98 (0.83; 1.15)	0.799	1.12 (0.91; 1.39)	0.277	0.92 (0.72; 1.18)	0.529
Hyperactivity	1.22 (1.04; 1.43)	**0.014**	1.09 (0.93; 1.28)	0.288	1.12 (0.93; 1.35)	0.223
Peer problems	1.13 (0.94; 1.37)	0.205	1.02 (0.85; 1.22)	0.851	1.12 (0.93; 1.35)	0.224
Prosocial	1.13 (0.92; 1.38)	0.257	1.02 (0.83; 1.25)	0.860	0.87 (0.68; 1.10)	0.248
**(B) Abnormal vs. normal/borderline**						
Total difficulties	1.25 (1.02; 1.53)	**0.031**	1.12 (0.67; 1.88)	0.672	1.04 (0.50; 2.15)	0.921
Emotional problems	1.26 (1.05; 1.52)	**0.013**	0.83 (0.59; 1.17)	0.289	0.88 (0.62; 1.25)	0.485
Conduct problems	1.00 (0.77; 1.28)	0.969	1.07 (0.78; 1.46)	0.683	0.98 (0.66; 1.45)	0.908
Hyperactivity	1.32 (1.09; 1.61)	**0.005**	0.98 (0.78; 1.23)	0.885	1.24 (0.95; 1.61)	0.111
Peer problems	1.09 (0.84; 1.40)	0.523	0.88 (0.57; 1.36)	0.562	1.20 (0.79; 1.84)	0.394
Prosocial	1.08 (0.77; 1.50)	0.658	0.97 (0.65; 1.43)	0.870	0.88 (0.57; 1.36)	0.571

^a^ OR and 95% CI modeled per interquartile range increase in PRAL (10-year cross-sectional = 14.9 mEq/d; 15-year cross-sectional = 17.8 mEq/d; prospective = 14.7 mEq/d); ^b^ Logistic regression models adjusted for sex, total energy intake, sedentary behavior, moderate-to-vigorous physical activity, BMI, pubertal status, parental education, study, and recruitment region; ^c^ Logistic regression models further adjusted for the respective mental health subscale assessed at age 10 years. Significant associations marked in bold: *p* < 0.05.

## Supplementary Materials

The following are available online at http://www.mdpi.com/2072-6643/10/5/582/s1, Table S1: OR and 95% CI assessing the cross-sectional and prospective associations of diet-induced acid load (PRAL) with mental health outcomes, additionally adjusting for parental psychopathology; Table S2: OR and 95% CI assessing the cross-sectional and prospective associations of diet-induced acid load (PRAL) with mental health outcomes, excluding participants with chronic diseases, Table S3: OR and 95% CI assessing the cross-sectional and prospective associations of diet-induced acid load (PRAL) with mental health outcomes, additionally adjusting for n-3 PUFA; Table S4: OR and 95% CI assessing the cross-sectional and prospective associations of diet-induced acid load (PRAL) with mental health outcomes, including only participants with complete data at both 10-year and 15-year follow-ups, Table S5: OR and 95% CI assessing the cross-sectional and prospective associations of diet-induced acid load (PRAL) with mental health outcomes defined using Lohbeck et al. cuf-offs for German adolescents; Table S6: Descriptive characteristics of study population with complete data for SDQ and FFQ at the 10-year and 15-year follow-ups; Figure S1: Distribution of PRAL in females and males, at the 10-year and 15-year follow-ups.
