# Supplementary material for: Dietary Acid Load and Mental Health Outcomes in Children and Adolescents: Results from the GINIplus and LISA Birth Cohort Studies
Source: Nutrients. 2018 May 8;10(5):582. doi: 10.3390/nu10050582 (PMC5986462; doi:10.3390/nu10050582)
Supplement: Supplementary file 1 [file nutrients-10-00582-s001.pdf]

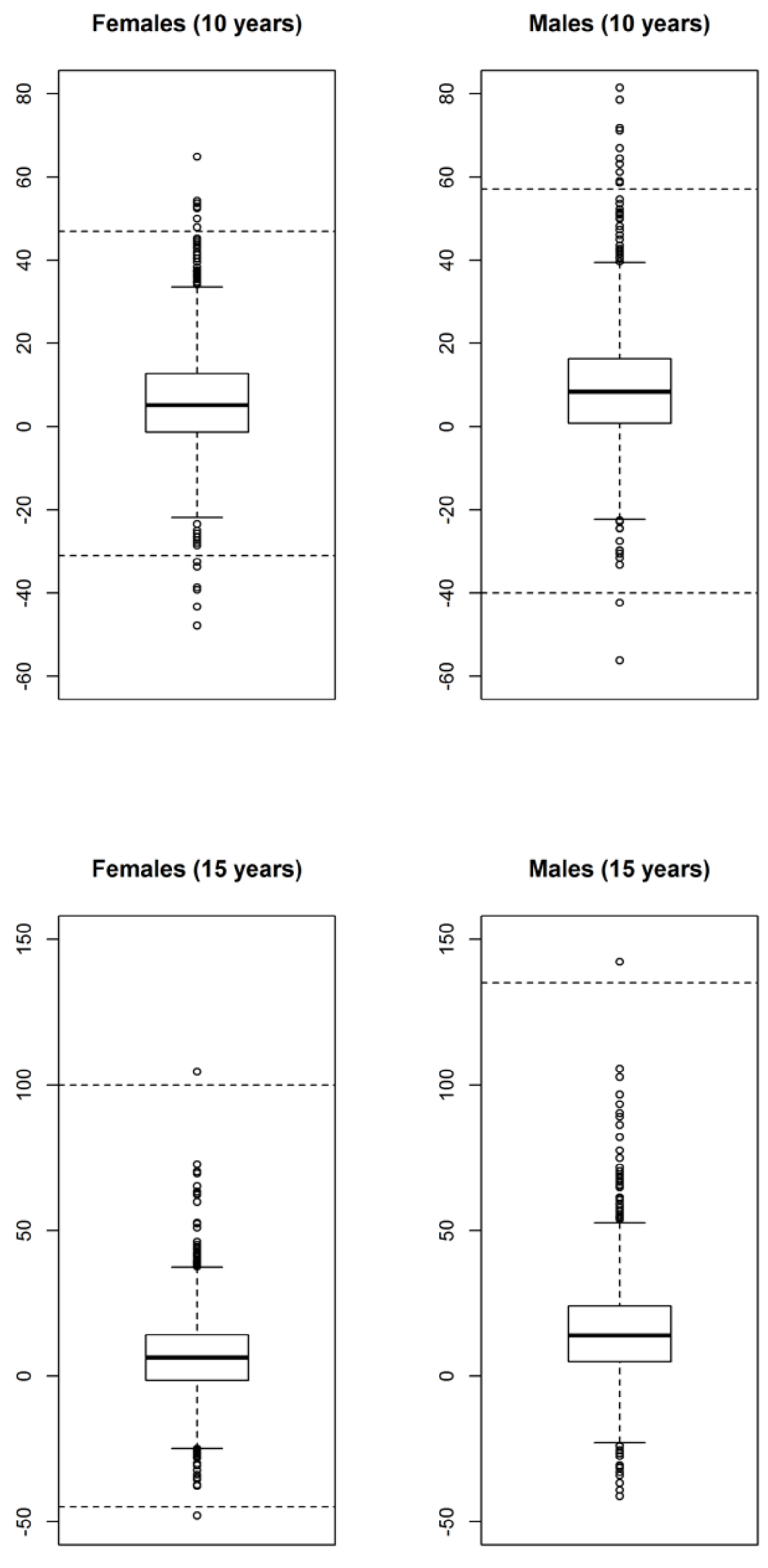

Figure S1. Distribution of PRAL in females (left) and males (right), at the 10- and 15-year follow-ups. The dotted lines indicate extreme values which were subsequently excluded from analyses

Table S1. OR and 95% CI assessing the cross-sectional and prospective associations of diet-induced acid load (PRAL) with mental health outcomes, additionally adjusting for parental psychopathology[a]

	Cross-sectional[b] (10-year Follow-up; N=2149)		Cross-sectional[b] (15-year Follow-up; N=2046)		Prospective[c] (10- to 15-year Follow-up; N=1674)	
**(A) Borderline/abnormal vs. normal**	OR (95%CI)	P-value	OR (95%CI)	P-value	OR (95%CI)	P-value
Total difficulties	1.22 (1.03;1.43)	**0.020**	1.05 (0.83;1.32)	0.691	0.93 (0.72;1.20)	0.590
Emotional problems	1.35 (1.16;1.58)	**<0.001**	1.06 (0.82;1.36)	0.662	1.04 (0.78;1.37)	0.807
Conduct problems	1.01 (0.85;1.20)	0.897	1.13 (0.91;1.40)	0.266	0.92 (0.72;1.18)	0.516
Hyperactivity	1.33 (1.12;1.58)	**0.001**	1.11 (0.94;1.31)	0.204	1.13 (0.94;1.36)	0.199
Peer problems	1.20 (0.98;1.46)	0.082	1.02 (0.85;1.22)	0.839	1.12 (0.92;1.35)	0.254
Prosocial	1.12 (0.90;1.40)	0.296	1.01 (0.82;1.24)	0.949	0.87 (0.68;1.10)	0.247
**(B) Abnormal vs. normal/borderline**	OR (95%CI)	P-value	OR (95%CI)	P-value	OR (95%CI)	P-value
Total difficulties	1.39 (1.11;1.72)	**0.003**	1.14 (0.68;1.91)	0.630	0.99 (0.47;2.06)	0.977
Emotional problems	1.28 (1.06;1.56)	**0.012**	0.85 (0.60;1.20)	0.353	0.89 (0.62;1.28)	0.531
Conduct problems	1.09 (0.84;1.42)	0.507	1.07 (0.77;1.47)	0.702	0.99 (0.66;1.47)	0.945
Hyperactivity	1.43 (1.16;1.77)	**0.001**	1.01 (0.80;1.27)	0.939	1.26 (0.97;1.64)	0.087
Peer problems	1.23 (0.93;1.62)	0.140	0.89 (0.57;1.37)	0.587	1.20 (0.78;1.83)	0.408
Prosocial	1.06 (0.76;1.50)	0.721	0.97 (0.66;1.44)	0.895	0.88 (0.57;1.35)	0.552

[a]OR and 95%CI modelled per interquartile range increase in PRAL (10-year cross-sectional=14.8 mEq/d; 15-year cross-sectional=17.8 mEq/d; prospective=14.8 mEq/d);
[b]Logistic regression models adjusted for sex, total energy intake, sedentary behaviour, moderate-vigorous physical activity, BMI, pubertal status, parental education, study, recruitment region and parental psychopathology; [c]Logistic regression models further adjusted for the respective mental health subscale assessed at age 10 years

Table S2. OR and 95% CI assessing the cross-sectional and prospective associations of diet-induced acid load (PRAL) with mental health outcomes, excluding participants with chronic diseases[a]

	Cross-sectional[b] (10-year Follow-up; N=2332)		Cross-sectional[b] (15-year Follow-up; N=2042)		Prospective[c] (10- to 15-year Follow-up; N=1669)	
**(A) Borderline/abnormal vs. normal**	OR (95%CI)	P-value	OR (95%CI)	P-value	OR (95%CI)	P-value
Total difficulties	1.13 (0.97;1.32)	0.128	0.98 (0.78;1.24)	0.891	0.91 (0.70;1.18)	0.468
Emotional problems	1.33 (1.15;1.54)	**<0.001**	0.99 (0.77;1.27)	0.934	1.01 (0.76;1.34)	0.947
Conduct problems	0.98 (0.83;1.16)	0.833	1.11 (0.90;1.37)	0.330	0.91 (0.71;1.17)	0.458
Hyperactivity	1.23 (1.05;1.44)	**0.011**	1.07 (0.91;1.26)	0.426	1.09 (0.90;1.31)	0.387
Peer problems	1.13 (0.94;1.37)	0.195	0.99 (0.83;1.19)	0.955	1.11 (0.92;1.34)	0.266
Prosocial	1.10 (0.89;1.35)	0.375	1.00 (0.81;1.22)	0.981	0.88 (0.69;1.12)	0.287
**(B) Abnormal vs. normal/borderline**	OR (95%CI)	P-value	OR (95%CI)	P-value	OR (95%CI)	P-value
Total difficulties	1.26 (1.03;1.54)	**0.027**	1.13 (0.67;1.90)	0.648	1.03 (0.50;2.15)	0.932
Emotional problems	1.26 (1.05;1.52)	**0.014**	0.82 (0.58;1.16)	0.259	0.88 (0.62;1.26)	0.488
Conduct problems	1.00 (0.78;1.28)	0.987	1.07 (0.78;1.46)	0.681	0.98 (0.66;1.46)	0.919
Hyperactivity	1.34 (1.10;1.63)	**0.004**	0.97 (0.77;1.22)	0.815	1.22 (0.93;1.58)	0.146
Peer problems	1.09 (0.84;1.41)	0.510	0.88 (0.56;1.37)	0.564	1.21 (0.79;1.85)	0.388
Prosocial	1.08 (0.78;1.50)	0.645	0.97 (0.66;1.43)	0.864	0.89 (0.57;1.38)	0.601

[a]OR and 95%CI modelled per interquartile range increase in PRAL (10-year cross-sectional=14.9 mEq/d; 15-year cross-sectional=17.6 mEq/d; prospective=14.8 mEq/d);
[b]Logistic regression models adjusted for sex, total energy intake, sedentary behaviour, moderate-vigorous physical activity, BMI, pubertal status, parental education, study, and recruitment region; [c]Logistic regression models further adjusted for the respective mental health subscale assessed at age 10 years

Table S3. OR and 95% CI assessing the cross-sectional and prospective associations of diet-induced acid load (PRAL) with mental health outcomes, additionally adjusting for n-3 PUFA

	Cross-sectional[b] (10-year Follow-up; N=2350)		Cross-sectional[b] (15-year Follow-up; N=2061)		Prospective[c] (10- to 15-year Follow-up; N=1685)	
**(A) Borderline/abnormal vs. normal**	OR (95%CI)	P-value	OR (95%CI)	P-value	OR (95%CI)	P-value
Total difficulties	1.11 (0.95;1.30)	0.184	1.01 (0.80;1.27)	0.929	0.93 (0.72;1.20)	0.566
Emotional problems	1.31 (1.14;1.52)	**<0.001**	1.02 (0.80;1.31)	0.864	1.02 (0.77;1.34)	0.900
Conduct problems	0.98 (0.83;1.16)	0.808	1.12 (0.91;1.39)	0.280	0.92 (0.72;1.18)	0.529
Hyperactivity	1.20 (1.02;1.41)	**0.026**	1.09 (0.93;1.29)	0.282	1.12 (0.93;1.35)	0.223
Peer problems	1.12 (0.93;1.36)	0.237	1.02 (0.85;1.22)	0.843	1.12 (0.93;1.35)	0.224
Prosocial	1.15 (0.93;1.41)	0.200	1.01 (0.83;1.24)	0.890	0.87 (0.68;1.10)	0.248
**(B) Abnormal vs. normal/borderline**	OR (95%CI)	P-value	OR (95%CI)	P-value	OR (95%CI)	P-value
Total difficulties	1.22 (1.00;1.49)	0.051	1.08 (0.64;1.81)	0.769	1.04 (0.50;2.15)	0.921
Emotional problems	1.25 (1.04;1.5)0	**0.019**	0.81 (0.58;1.14)	0.234	0.88 (0.62;1.25)	0.485
Conduct problems	1.00 (0.78;1.29)	0.986	1.08 (0.78;1.48)	0.647	0.98 (0.66;1.45)	0.908
Hyperactivity	1.30 (1.07;1.58)	**0.008**	0.98 (0.78;1.23)	0.867	1.24 (0.95;1.61)	0.111
Peer problems	1.08 (0.84;1.40)	0.552	0.87 (0.56;1.34)	0.515	1.20 (0.79;1.84)	0.394
Prosocial	1.09 (0.78;1.52)	0.608	0.98 (0.66;1.46)	0.923	0.88 (0.57;1.36)	0.571

[a]OR and 95%CI modelled per interquartile range increase in PRAL (10-year cross-sectional=14.9 mEq/d; 15-year cross-sectional=17.8 mEq/d; prospective=14.7 mEq/d);
[b]Logistic regression models adjusting for sex, total energy intake, sedentary behaviour, moderate-vigorous physical activity, BMI, pubertal status, parental education, study, recruitment region, and n-3 PUFA; [c]Logistic regression models further adjusting for the respective mental health subscale assessed at age 10 years

Table S4. OR and 95% CI assessing the cross-sectional and prospective associations of diet-induced acid load (PRAL) with mental health outcomes, including only participants with complete data at both 10- and 15-year follow-ups[a]

	Cross-sectional[b] (10-year Follow-up; N=1146)		Cross-sectional[b] (15-year Follow-up; N=1146)		Prospective[c] (10- to 15-year Follow-up; N=1146)	
**(A) Borderline/abnormal vs. normal**	OR (95%CI)	P-value	OR (95%CI)	P-value	OR (95%CI)	P-value
Total difficulties	1.06 (0.85;1.32)	0.632	1.12 (0.81;1.56)	0.482	0.88 (0.61;1.26)	0.474
Emotional problems	1.39 (1.12;1.71)	**0.003**	1.06 (0.74;1.51)	0.768	0.91 (0.63;1.31)	0.595
Conduct problems	1.01 (0.80;1.28)	0.904	0.84 (0.62;1.13)	0.250	0.79 (0.57;1.08)	0.141
Hyperactivity	1.19 (0.93;1.50)	0.163	1.06 (0.85;1.32)	0.615	1.20 (0.93;1.55)	0.169
Peer problems	1.13 (0.86;1.49)	0.376	1.06 (0.84;1.33)	0.613	1.13 (0.88;1.46)	0.327
Prosocial	1.04 (0.77;1.39)	0.811	1.07 (0.79;1.45)	0.654	0.85 (0.62;1.18)	0.331
**(B) Abnormal vs. normal/borderline**	OR (95%CI)	P-value	OR (95%CI)	P-value	OR (95%CI)	P-value
Total difficulties	1.06 (0.79;1.42)	0.700	2.35 (1.03;5.37)	**0.042**	1.88 (0.55;6.40)	0.310
Emotional problems	1.22 (0.94;1.59)	0.137	0.91 (0.58;1.41)	0.668	0.79 (0.50;1.24)	0.311
Conduct problems	1.14 (0.80;1.62)	0.475	0.82 (0.51;1.33)	0.419	0.91 (0.56;1.50)	0.722
Hyperactivity	1.27 (0.95;1.70)	0.108	1.01 (0.73;1.41)	0.936	1.64 (1.12;2.39)	**0.011**
Peer problems	1.19 (0.81;1.73)	0.377	0.74 (0.40;1.35)	0.320	0.95 (0.48;1.87)	0.873
Prosocial	0.93 (0.58;1.48)	0.747	1.02 (0.62;1.68)	0.938	1.03 (0.60;1.76)	0.927

[a]OR and 95%CI modelled per interquartile range increase in PRAL (10-year cross-sectional and prospective=14.2 mEq/d; 15-year cross-sectional=16.8 mEq/d); [b]Logistic regression models adjusting for sex, total energy intake, sedentary behaviour, moderate-vigorous physical activity, BMI, pubertal status, parental education, study, and recruitment region; [c]Logistic regression models further adjusting for the respective mental health subscale assessed at age 10 years

Table S5. OR and 95% CI assessing the cross-sectional and prospective associations of diet-induced acid load (PRAL) with mental health outcomes defined using Lohbeck et al. cuf-offs for German adolescents[a]

**1. Including maximal available data at 10 and 15 years**	Cross-sectional[b] (15-year Follow-up; N=2061)		Prospective[c] (10- to 15-year Follow-up; N=1685)	
**(A) Borderline/abnormal vs. normal**	OR (95%CI)	P-value	OR (95%CI)	P-value
Total difficulties	1.01 (0.83;1.23)	0.909	1.05 (0.85;1.30)	0.665
Emotional problems	0.95 (0.81;1.12)	0.545	0.96 (0.80;1.14)	0.620
Conduct problems	1.04 (0.90;1.19)	0.597	0.95 (0.82;1.11)	0.535
Hyperactivity	1.08 (0.95;1.22)	0.250	1.00 (0.87;1.16)	0.970
Peer problems	1.02 (0.85;1.22)	0.851	1.12 (0.93;1.35)	0.224
Prosocial	1.04 (0.87;1.24)	0.674	0.91 (0.74;1.12)	0.357
**(B) Abnormal vs. normal/borderline**	OR (95%CI)	P-value	OR (95%CI)	P-value
Total difficulties	1.05 (0.74;1.48)	0.798	1.00 (0.68;1.48)	0.996
Emotional problems	0.92 (0.68;1.24)	0.579	0.93 (0.66;1.31)	0.690
Conduct problems	1.06 (0.81;1.37)	0.678	1.01 (0.76;1.36)	0.929
Hyperactivity	0.98 (0.78;1.23)	0.885	1.24 (0.95;1.61)	0.111
Peer problems	0.94 (0.66;1.33)	0.732	1.17 (0.82;1.65)	0.384
Prosocial	1.05 (0.78;1.42)	0.745	0.84 (0.59;1.20)	0.338
**2. Only including complete data at both 10 and 15 years**	**Cross-sectional[b] (15-year Follow-up; N=1146)**		**Prospective[c] (10- to 15-year Follow-up; N=1146)**	
**(A) Borderline/abnormal vs. normal**	OR (95%CI)	P-value	OR (95%CI)	P-value
Total difficulties	1.08 (0.82;1.42)	0.600	0.99 (0.73;1.34)	0.961
Emotional problems	0.91 (0.72;1.15)	0.409	0.87 (0.69;1.11)	0.258
Conduct problems	0.99 (0.82;1.19)	0.893	0.90 (0.74;1.10)	0.299
Hyperactivity	1.12 (0.94;1.33)	0.212	0.99 (0.82;1.19)	0.918
Peer problems	1.06 (0.84;1.33)	0.613	1.13 (0.88;1.46)	0.327
Prosocial	1.11 (0.85;1.45)	0.437	0.81 (0.61;1.08)	0.148
**(B) Abnormal vs. normal/borderline**	OR (95%CI)	P-value	OR (95%CI)	P-value
Total difficulties	1.35 (0.84;2.16)	0.218	0.98 (0.55;1.74)	0.945
Emotional problems	0.91 (0.59;1.40)	0.667	0.87 (0.56;1.35)	0.529
Conduct problems	0.79 (0.54;1.16)	0.236	0.82 (0.56;1.20)	0.302
Hyperactivity	1.01 (0.73;1.41)	0.936	1.64 (1.12;2.39)	**0.011**
Peer problems	0.94 (0.60;1.48)	0.781	1.09 (0.64;1.86)	0.747
Prosocial	1.12 (0.74;1.69)	0.590	0.98 (0.62;1.54)	0.920

[a]OR and 95%CI modelled per IQR increase in PRAL (1. Including maximal data available: 15-year cross-sectional=17.8 mEq/d; prospective =14.7 mEq/d. 2. Including complete data: 15-year cross-sectional=16.8 mEq/d; prospective =14.2 mEq/d.); [b]Logistic regression models adjusted for sex, total energy intake, sedentary behaviour, moderate-vigorous physical activity, BMI, pubertal status, parental education, study, and recruitment region; [c]Logistic regression models further adjusted for the respective mental health subscale assessed at age 10 years

Table S6. Descriptive characteristics of study population with complete data for SDQ and FFQ at the 10- and 15-year follow-ups

	10-year Follow-up				15-year Follow-up			
	Total (N=1146)	Females (N=589)	Males (N=557)	P-value[a]	Total (N=1146)	Females (N=589)	Males (N=557)	P-value[a]
**Total difficulties** [borderline]	75 (6.54)	26 (4.41)	49 (8.80)	**0.002**	51 (4.45)	32 (5.43)	19 (3.41)	0.160
[abnormal]	79 (6.89)	34 (5.77)	45 (8.08)		9 (0.79)	6 (1.02)	3 (0.54)	
**Emotional problems** [borderline]	77 (6.72)	44 (7.47)	33 (5.92)	0.340	24 (2.09)	22 (3.74)	2 (0.36)	**<0.001**
[abnormal]	106 (9.25)	49 (8.32)	57 (10.23)		40 (3.49)	35 (5.94)	5 (0.90)	
**Conduct problems** [borderline]	79 (6.89)	27 (4.58)	52 (9.34)	**<0.001**	44 (3.84)	19 (3.23)	25 (4.49)	**0.001**
[abnormal]	50 (4.36)	18 (3.06)	32 (5.75)		25 (2.18)	4 (0.68)	21 (3.77)	
**Hyperactivity** [borderline]	48 (4.19)	19 (3.23)	29 (5.21)	**<0.001**	74 (6.46)	39 (6.62)	35 (6.28)	0.096
[abnormal]	84 (7.33)	24 (4.07)	60 (10.77)		54 (4.71)	20 (3.40)	34 (6.10)	
**Peer problems** [borderline]	44 (3.84)	24 (4.07)	20 (3.59)	0.370	106 (9.25)	46 (7.81)	60 (10.77)	0.210
[abnormal]	50 (4.36)	21 (3.57)	29 (5.21)		18 (1.57)	10 (1.70)	8 (1.44)	
**Prosocial** [borderline]	57 (4.97)	20 (3.40)	37 (6.64)	**0.005**	50 (4.36)	14 (2.38)	36 (6.46)	**0.002**
[abnormal]	34 (2.97)	12 (2.04)	22 (3.95)		27 (2.36)	11 (1.87)	16 (2.87)	
**PRAL** (mEg/d))	6.14 (-0.37; 13.87)	4.51 (-1.45; 11.68)	8.33 (0.80; 15.4)	**<0.001**	9.63 (1.68; 18.51)	6.15 (-1.05; 13.64)	13.9 (5.50; 23.30)	**<0.001**
**BMI** (kg/m^2)	16.6 (15.5; 18.2)	16.6 (15.5; 18.2)	16.6 (15.5; 18.3)	0.543	20 (18.6; 21.7)	20.1 (18.7; 21.7)	19.9 (18.5; 21.7)	0.151
**Total energy intake** (kcal/day)	1892 (1573; 2255)	1780 (1487; 2107)	2066 (1673; 2405)	**<0.001**	1994 (1569; 2504)	1724 (1357; 2127)	2284 (1857; 2784)	**<0.001**
**Moderate-vigorous PA** [low]	252 (21.99)	158 (26.83)	94 (16.88)	**<0.001**	263 (22.95)	166 (28.18)	97 (17.41)	**<0.001**
[medium]	631 (55.06)	328 (55.69)	303 (54.40)		621 (54.19)	324 (55.01)	297 (53.32)	
[high]	263 (22.95)	103 (17.49)	160 (28.73)		262 (22.86)	99 (16.81)	163 (29.26)	
**Screen-time** [high]	103 (8.99)	46 (7.81)	57 (10.23)	0.180	606 (52.88)	256 (43.46)	350 (62.84)	**<0.001**
**Puberty signs** [yes]	347 (30.28)	278 (47.20)	69 (12.39)	**<0.001**	-	-	-	
**Pubertal stage** [pre-mid]	-	-	-		265 (23.12)	28 (4.75)	237 (42.55)	**<0.001**
[late]	-	-	-		784 (68.41)	468 (79.46)	316 (56.73)	
[post]	-	-	-		97 (8.46)	93 (15.79)	4 (0.72)	

	Basic covariates (same for 10- and 15-year follow-up)			
	Total (N=2350)	Females (N=1137)	Males (N=1213)	P-value[a]
**Parental education** [high]	847 (73.91)	450 (76.40)	397 (71.27)	0.056
**Study (arm)** [GINI (observation)]	418 (36.47)	231 (39.22)	187 (33.57)	**0.024**
[GINI (intervention)]	304 (26.53)	162 (27.50)	142 (25.49)	
[LISA]	424 (37.00)	196 (33.28)	228 (40.93)	
**Region** [Munich]	650 (56.72)	331 (56.20)	319 (57.27)	0.460
[Leipzig]	80 (6.98)	37 (6.28)	43 (7.72)	
[Bad Honnef]	54 (4.71)	25 (4.24)	29 (5.21)	
[Wesel]	362 (31.59)	196 (33.28)	166 (29.80)	

Values are presented as counts (%) for categorical variables, and medians (25th; 75th percentile) for continuous variables.

[a]Comparison between males and females: tested by Wilcoxon's rank-sum test for continuous variables, and by Pearson's $\chi 2$ test for categorical variables

Moderate-vigorous PA = Moderate-vigorous physical activity
